# Effects of *Lacticaseibacillus rhamnosus* HN001 on Happiness and Mental Well-Being: Findings from a Randomized Controlled Trial

**DOI:** 10.3390/nu16172936

**Published:** 2024-09-02

**Authors:** Imad Al Kassaa, Maher Fuad

**Affiliations:** Fonterra Research and Development Centre, Dairy Farm Road, Palmerston North 4442, New Zealand; imad.alkassaa@fonterra.com

**Keywords:** *Lacticaseibacillus rhamnosus* HN001, probiotics, psychobiotics, stress management, happiness, randomized controlled trial, mental health, mental wellness, mental well-being

## Abstract

Background/Objectives: *Lacticaseibacillus rhamnosus* HN001 (HN001) is a probiotic strain widely studied for its potential to improve human health. Previous studies have demonstrated promising results for HN001 in the improvement of mental well-being, particularly in terms of increased happiness and support for stress management in healthy adults. Methods: To further explore these findings, a double-blind, placebo-controlled trial was conducted with 120 participants aged ≥ 18 years with mild to high stress measured by the Perceived Stress Scale (PSS). The participants were randomly assigned to receive either HN001 or placebo for 28 days. Psychological assessments, including the Oxford Happiness Questionnaire (OHQ), were completed at baseline, day 14, and day 28. Secondary outcomes included changes in PSS scores, as well as depression, anxiety, stress, and total score levels measured by the DASS-21 questionnaire. Results: While not statistically significant, participants who received HN001 showed an improvement in OHQ (mean change, 13.3) and PSS total scores (mean change, −8.1) over time compared with the placebo group (mean change, 10.2 and −6.6, respectively). Furthermore, 39% of the participants moved from not happy to happy, compared with only 29% in the placebo group. Post-hoc analysis showed a statistically significant interaction between intervention and study day for OHQ and PSS total scores, with *p*-values of 0.014 and 0.043, respectively. No adverse effects were observed. Conclusions: HN001 showed improvements in both happiness and PSS scores. Furthermore, sex subgroup analysis revealed statistically significant differences in both outcomes, emphasizing the need for larger and longer intervention studies.

## 1. Introduction

Over the past few decades, there has been growing interest in the role of the gut microbiome in brain health, mental wellness, and overall well-being. The American Psychological Association (APA) defines “well-being” as a state of happiness and contentment, with low levels of distress, overall good physical and mental health, and outlook, or good quality of life [1]. Many studies have shown an association between a balanced, healthy gut microbiota and a reduction in stress, depression, and anxiety, as well as improved cognition, memory, and other brain health outcomes [2].

The enteric nervous system (ENS) comprises nearly 100 million neurons that can function autonomously, and interact with the human digestive system [3]. Dietary components and digestive by-products interact with the ENS, which in turn communicates with the central nervous system (CNS). Furthermore, microbial metabolites can have a significant impact on gut physiology, immunity, and ENS stimulation. Hence, the microbiota-gut-brain axis has been described to emphasize the role of the gut microbiota in gut- brain interactions [3]. Indeed, it appears that the gut microbiota can maintain good communication with the CNS via at least two different routes: the ENS and humoral immunity. Microbial metabolites can stimulate the ENS by direct interaction with gut neurons or indirectly by initiating a cascade of host responses that ultimately modulate ENS responses. Additionally, immune signalling molecules (e.g., cytokines and chemokines) [4] and endocrine messages (e.g., cortisol and norepinephrine (NE) [5]) can be influenced by both gut microbes and their metabolites (e.g., short-chain fatty acids: SCFA) [6,7]. These metabolites may travel to the brain via the blood and cross the blood-brain barrier (BBB) [8].

The microbiota-gut-brain axis has been supported by many studies that have reported differences in the gut microbiota composition between healthy subjects and patients with mental health disorders [9,10]. In addition, studies have emphasized the importance of key microbial taxa, particularly the SCFA producers, to maintain brain health and manage stress, anxiety, and depression. These studies showed that some microbial species may contribute to the production of neurotransmitters and SCFA that impact brain physiology and enhance brain function [6,7,8].

*Lacticaseibacillus rhamnosus* HN001 (*Lb. rhamnosus* HN001; formerly named *Lactobacillus rhamnosus* HN001 and variously trademarked as DR20™ and HN001™ is one of the most well-known probiotic strains and has been extensively studied in the past two decades. Apart from its immune-enhancing benefits [11], *Lb. rhamnosus* HN001 has also shown efficacy in the areas of gut health [12], skin allergy [13], and gestational diabetes [14], as well as in postpartum depression [15], mental health, and social functioning [16].

In three different clinical and two preclinical studies, HN001 was selected to explore its potential effects on mental well-being and brain health. HN001 showed a positive effect on social functioning and anxiety in prediabetes patients [16], lowered anxiety and depression postpartum [15], and improved total happiness scores in a consumer experience study carried out in 2022 [17]. In addition, HN001 showed noticeable effects on rats’ psychological-like behaviors and brain function via immune modulation mechanism as well as normalizing the level of blood neurotransmitters [18,19].

A recent study by Lonstein et al. [20] demonstrated that *Lb. rhamnosus* HN001 reduced postpartum anxiety-related behaviors in rodent models by maintaining neurotransmitter balance (NE, serotonin, dopamine) in pregnant rat brains.

Over the years, the prevalence of mental health issues and disability-adjusted life years (DALYs) attributed to mental health have been increasing [21]. The COVID-19 pandemic is likely to have exacerbated the issue, and more people have started to suffer with their mental health [22] and reduced happiness [23]. The objective of the current study was to investigate the role of HN001 on mental wellness, specifically the happiness aspect of mental wellness measured by happiness scores over a period of 28 days.

## 2. Materials and Methods

### 2.1. Study Design

This study was conducted on healthy men and women ≥ 18 years old with no current diagnosis of mental health disorders or use of related mental health medications. Participant selection was based on pre-screening using the Perceived Stress Scale (PSS) questionnaire, and subjects who scored ≥14, indicating moderate to high stress levels, were selected for further screening. This study was a decentralized, randomized, double-blind, placebo-controlled, parallel design with one screening and randomization visit followed by a 27-day (±2 days) intervention period with outcomes assessed at Day 0 (baseline: T0), Day 13 (midpoint: T1), and Day 30 (end time: T2).

The study was conducted in compliance with the ethical principles based on the Declaration of Helsinki (2013), Title 21 of the Code of Federal Regulations §§ 50, 54, 56, and 312, International Conference on Harmonisation E6, and standards of Good Clinical Practice. The initial study protocol was approved by the central Institutional Review Board (IRB), Sterling IRB, on 1 June 2023, before study commencement. A protocol clarification memo, revised participant informed consent form, and authorization to use and disclose medical information were approved on 19 June 2023, reference 11036-DBBeckman. This trial was also registered in the clinicaltrial.gov database under the registration number NCT05905679. Signed written informed consent for participation in the study was obtained from all participants before protocol-specific procedures were carried out. Participants were informed of their right to withdraw from the study at any time. The study was explained verbally, as well as on the IRB-approved informed consent form.

The study’s primary outcome was the happiness total score derived from the Oxford Happiness Questionnaire (OHQ) at the end of the study (T2). The secondary outcomes were the OHQ total score (at T1), Perceived Stress Scale (PSS) total score, depression, anxiety, and stress scale (DASS-21) subscales, and total scores at T1 and T2, as well as the change from T0 to each endpoint (T1 and T2).

### 2.2. Participants

**Figure 1:**
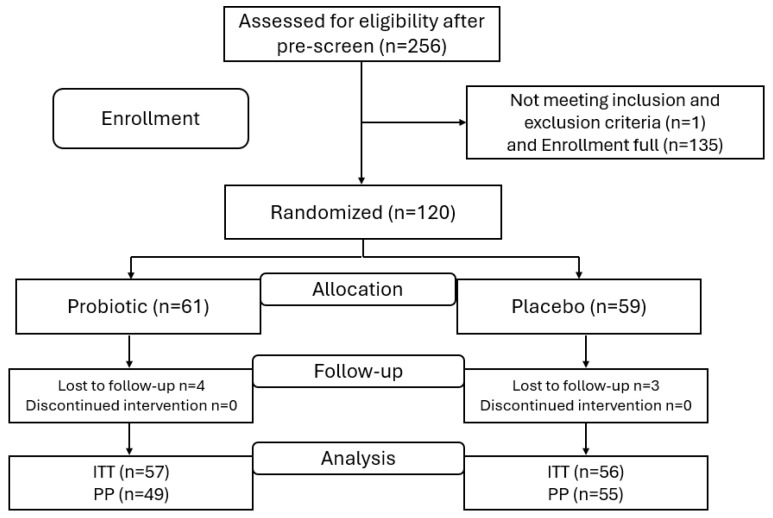
Study flow chart; *n*, number of participants; ITT, intent-to-treat; PP, per protocol.

This study consisted of a probiotic arm (*n* = 59) and a placebo arm (*n* = 61). The intent-to-treat (ITT) population included all randomized participants (*n* = 120) (Figure 1). The per protocol (PP) population is a subset of the ITT that completed the study in full compliance (*n* = 104). The reasons for exclusion from the PP were (i) product non-compliance (*n* = 8) and (ii) not completing the OHQ questionnaire (*n* = 8). The participants’ characteristics are detailed in Table 1.

Inclusion criteria consisted of age ≥ 18, access to a smartphone, and willingness to refrain from supplements and products that might influence the study’s outcomes. Participants were then further selected as those with a PSS-10 score ≥ 14 (mild- to high-stress level) [24]. Exclusion criteria included: clinically diagnosed with neurologic or psychiatric disorders and currently requiring or under neurological/psychological/antibiotics treatment; health conditions such as diagnosed gastrointestinal conditions that would potentially interfere with the evaluation of the study product (e.g., inflammatory bowel disease, irritable bowel syndrome, Crohn’s disease, celiac disease); use of products that could influence the study’s outcomes (cannabis, marijuana or cannabinoid products) within 6 months of screening; consuming biotics products (pre-, pro- and post-biotics) within 2 weeks of screening; pregnancy or planning to be pregnant during the study period; lactating; unwilling to commit to the use of a medically approved form of contraception throughout the study period; recent history of (within 12 months of screening) or strong potential for alcohol or substance abuse; and any other condition that the Principal Investigator believed would interfere with the participant’s ability to provide informed consent, comply with the study protocol, confound the interpretation of the study results, or put the participant at undue risk.

### 2.3. Randomization and Blinding Protocol

Participants were enrolled with a target distribution of approximately 50% males and 50% females as per biological sex. Randomization of the two intervention groups was 1:1 stratified by biological sex and was performed using the randomization module of the Castor platform (Castor, New York, NY, USA) following a variable block model of sizes 4 and 6. The randomized sequence and codes were generated by Castor, and product labeling was conducted at the Fonterra Research and Development Center (FRDC) by independent employees who were not involved in the study. The labeled products were sent anonymously to the clinical trial provider.

The randomization number and assigned intervention group were recorded in each participant’s source documentation. Participants and study staff were blinded to the study product throughout the study. A set of sealed envelopes was provided to the clinical investigator for use in an emergency situation, where knowledge of the assignment was essential for the subjects’ immediate medical care.

### 2.4. Study Protocol

As represented in Figure 1, the study protocol consisted of 4 phases. The enrollment phase was preceded by an assessment of participant eligibility based on the exclusion and inclusion criteria. The second phase was the product allocation based on an independent randomization process with a robust blinding protocol. The third phase was the intervention stage, in which participants were instructed to consume the assigned capsules daily for over 28 days with a 3-day window. During the intervention, the active group was assigned Nutiani HN001™ capsules (supplied by Fonterra Cooperative Group, Auckland, New Zealand) (3 per day), providing a dose of 6 × 10^9^ CFU day^−1^ of *Lb. rhamnosus* HN001. The control group received a placebo (maltodextrin) with the same number of capsules per day. Participants received an e-contact through the Castor electronic platform during the intervention period at T0 (the first day of product consumption), T1 (midpoint), and T2 (the end of the intervention) to complete the questionnaires. Adverse events were assessed at the end of each e-contact. Participants were sent notifications and links through the Castor Connect app to complete all questionnaires at each time point throughout the intervention period and to complete the daily study product log. The final measure of compliance was estimated as the amount of returned unused study products collected at the end of the study, as a percentage of the scheduled intake. At the end of the intervention phase, the last phase consisted of data collection and statistical analyses.

### 2.5. Efficacy Parameters

Electronic-based questionnaires accessible via smartphones were used to simplify and encourage participants [25] to complete the questionnaires on time. The Oxford Happiness Questionnaire (OHQ) is a widely used psychological assessment tool designed to measure an individual’s subjective happiness [26]. The OHQ used in this study, including the short form, was validated and showed consistency and reliability across different studies [27]. The questionnaire was composed of 29 statements that respondents rated based on how well each statement described their personal feelings and experiences. The sum of the item scores is an overall measure of happiness, with higher scores indicating greater happiness. The total score was categorized using the following cutoffs: happy (score ≥ 4) versus not happy (score < 4).

Cohen’s Perceived Stress Scale (PSS) is a recognized questionnaire for measuring the perception of stress [28]. The PSS is a retrospective 10-item questionnaire that measures feelings and thoughts “over the past month” [28]. Scores were obtained by reversing the responses to the four positively stated items (questions 4, 5, 7, and 8) and then summing across all items. The PSS total score was categorized as follows: a score range of 0 to 13 represented a low level of stress, 14 to 26 represented moderate stress, and 27 to 40 represented a high level of stress.

Depression Anxiety Stress Scale-21 (DASS-21) was administered at T0, T1, and T2. This questionnaire is a shorter version of the original 42-item questionnaire, both of which were designed to evaluate depression, anxiety, and stress [29]. The DASS-21 has 21 items in 3 subscales of 7 items each. Response options are on a 4-point scale (0 = did not apply to me at all; 3 = applied to me most of the time). Each subscale score was calculated as the sum of the respective 7 items multiplied by 2. Scores range from 0 to 42, with higher scores indicating more psychological distress.

### 2.6. Statistical Analyses

Statistical programming and analyses were performed using SAS^®^ (SAS Institute, Inc., Cary, NC, USA), version 9.4, and/or figures created with R (R Core Team 2022, Vienna, Austria) version 4.2.2.

The study is sufficiently powered with 84 participants, assuming a moderate effect size of f = 0.25, power of 80%, a significance level of 0.05, 3 measurements, and a correlation between measurements of 0.5. Given the nature of the research being an online survey, where we expected more dropouts or loss-to-follow-up to be higher than the standard, attrition was calculated as 28%. Therefore, 120 (approximately 60 in each group) participants were enrolled. At the end of the study, 113 participants completed the study (response rate 94%). The primary analysis was conducted on the intent-to-treat population (*n* = 113).

A repeated measures model was used to assess the response profile for the total score of the OHQ, PSS, and DASS-21. The model included terms for the intervention groups on the study day (at T0, T1, and T2), the interaction between the intervention group and study day, and biological sex. Note that the proportion is calculated from the number of participants responding to the questionnaire at a given time point. The unstructured covariance structure was chosen so that the Akaike Information Criterion corrected (AICc) was minimized. Model-derived means, accompanied by 95% confidence intervals (CI), were calculated to estimate differences between intervention groups at each post-randomization time point, as well as the change from baseline to each post-randomization time point within each intervention group. The unadjusted *p*-value was presented, as well as the Holm (stepdown Bonferroni) adjusted *p*-value.

All post-hoc subgroup analyses were conducted within the biological sex subgroups. The analyzed outcomes included all questionnaires used in this study.

## 3. Results

### 3.1. Unless Otherwise Specified, Results Are Presented for the ITT Population, with Any Differences from the PP Population Noted

Participant details are listed in Table 1. Briefly, overall, the median age was 48 years, with an approximately equal distribution of male and female participants. The mean age (SD) for the probiotic group was 46.6 (15.8) years, which was similar to that of the placebo group [45.3 years (14.5) years].

### 3.2. OHQ Results

HN001 Supplementation Increases Happiness with Significant Sex-Specific Effects. Happiness score results are shown in Table 2. Model-derived estimates are presented in Supplementary Materials Figure S1. No significant differences were detected between the groups at T1 and T2 or the change from T0 to T1 and T2 (*p* > 0.05). The total happiness score increased from T0 to T2 in the probiotic group (mean change, 13.3; 95% CI, 8.8 to 17.8), whereas the placebo group showed an increase in the total happiness score (mean change, 10.2; 95% CI, 5.7 to 14.8) that was relatively lower than the probiotic group. The results were consistent in the PP population.

The categorized total happiness score distribution showed that 39 participants (65%) in the probiotic group (39 out of 60 participants reported at T2) reported being happy compared with 36 out of 59 (61%) in the placebo group. Interestingly, of the 56 participants who reported at T0 and T2 in the probiotic group, 22 (39%) moved from not happy to happy, compared with only 16 (29%) in the placebo group (Supplementary Materials Figure S2).

Post-hoc subgroup analyses were conducted to examine the potential effect of HN001 intake between males and females (Table 3). For the total happiness score for female participants, the results showed no significant interaction, no significant differences between groups at the respective post-baseline time points, or a change from baseline to post-baseline time points. However, in the male subgroup, a significant effect was detected for the intervention group by day interaction (*p*-value, 0.014) for OHQ total score, with an estimated partial eta2 of 0.05 (effect size f = 0.23) and estimated omega2 of 0.04 (effect size f = 0.20). This indicates that group impact on total happiness score was dependent on the study day, likely driven by the change in slope between T1 and T2 endpoints (i.e., the score continues to increase from T1 to T2 while the placebo group score appeared to plateau, resulting in the lines crossing; see Supplementary Materials Figure S3). However, no statistically significant differences were detected between the groups in the pre-specified comparisons of interest at each post-baseline day or the change from baseline to the post-baseline endpoints (*p* > 0.05). While the total happiness score increased from T0 to T2 in the probiotic group [11.1 (95% CI, 5.1 to 17.2)], the confidence interval for the placebo group contained zero [5.0 (95% CI, −1.3 to 11.3)] indicating a non-significant change from baseline (T0).

The distribution of the categorized happiness score showed that of the 30 female probiotic participants completing the questionnaire at T2, 21 (70%) reported being happy compared with 17 of 30 (57%) placebo participants. On the other hand, 12 out of 26 (46%) female participants (completing both T0 and T2 questionnaires) in the probiotic group moved from not happy to happy, whereas 8 out of 28 (29%) female participants in the placebo group moved from not happy to happy (Supplementary Materials Figure S4). Of the 30 male probiotic participants completing the questionnaire at T2, 18 (60%) reported being happy compared with 19 out of 29 (65%) placebo participants. Interestingly, of the 28 placebo participants completing both T0 and T2 questionnaires, 8 (29%) moved from not happy to happy. Of the 30 probiotic participants, 10 (33%) moved from not happy to happy (Supplementary Materials Figure S4).

### 3.3. Enhanced Perceived Stress Reduction with HN001 Supplementation and Significant Sex-Specific Effects

The impact of probiotic intake on participants’ stress levels was assessed using the PSS questionnaire. The results indicated a lack of significant interaction between the intervention group and study days. Additionally, no significant differences were observed between the groups at any post-randomization time point or in the change from baseline to each intervention time point (Table 3). The biggest change in the stress score in the HN001 group (from T0 to T2) was [−8.1 (95% CI, −10.3 to −5.9)] compared with the placebo group, which was [−6.6 (95% CI, −8.8 to −4.3)] as shown by Supplementary Materials Figure S5a. Moreover, the distribution of categorized PSS scores showed that at T2, 28 of 60 HN001 participants (47%) reported low stress compared with 23 of 59 (39%) placebo participants (Supplementary Materials Figure S6).

Similar to OHQ post-hoc analyses, PSS scores in the female subgroup showed no significant differences in all different analysis types (*p*-value > 0.05). However, the PSS score in the male subgroup showed a statistically significant interaction between intervention and study day (*p*-value, 0.043) with an estimated partial eta2 of 0.0363 (effect size f = 0.194) and estimated omega2 of 0.0208 (effect size f = 0.146) (Supplementary Materials Figure S5b). The PSS scores for the male subgroup were consistent with the results of the total happiness score. The distribution of the categorized PSS total score by sex is presented in Supplementary Materials Figure S7. Twelve of the 30 (40%) female probiotic participants reported low stress at T2 compared to 10 of the 30 (33%) placebo participants. On the other hand, 16 of 30 (53%) male probiotic participants reported low stress at T2 compared with 13 of 29 (45%) placebo participants.

### 3.4. No Significant Changes in DASS-21 Scores

The DASS-21 questionnaire results showed no significant results for anxiety, depression, or stress as well as the DASS-21 total score for all measurement types, the interaction between the intervention group and days, group differences at each post-randomization point, and the change from the baseline to each post-randomization point (*p*-value > 0.05) (Table 3). Both intervention groups showed changes in the DASS-21 components from baseline (T0) to T2. For instance, anxiety score [−3.6 (95% CI, −5.2 to −2.0)], depression [−4.9 (95% CI, −6.7 to −3.1)], and total score [−14.6 (95% CI, −19.1 to −10.1)] showed decreases in both probiotics and placebo groups scores [mean changes: −2.9 (95% CI, −4.5 to −1.3), (−3.2 (95% CI, −5.0 to −1.3), and −12.4 (95% CI, −16.9 to −7.9), respectively].

The distribution of categorized DASS-21 subscale scores showed that at T2, 67% of probiotic participants (40 of 60), compared with 64% of placebo participants (38 of 59), had normal depression; 47 (78%) probiotic participants and 44 (75%) placebo participants had normal anxiety, and 41 (68%) and 37 (62%) had normal anxiety for the probiotic and placebo groups, respectively.

Regarding the DASS-21 post-hoc analysis results, both subgroups showed no statistical significance in all analysis types (Table 3). However, the same improvement was found in the three DASS-21 components (anxiety, depression, and total score). For instance, anxiety marginally decreased in the probiotic group [mean change −1.9 (95% CI, −3.7 to −0.1)], while the confidence interval for the placebo group contained zero [−0.9 (95% CI, −2.8 to 1.0)]. Similarly, depression decreased from T0 to T2 in the probiotic group [−4.5 (95% CI, −6.9 to −2.1)], but the confidence interval contained zero for the placebo group [−2.1 (95% CI, −4.7 to 0.4)]. On the other hand, the stress score decreased from T0 to T2 in the placebo [−4.1 (95% CI, −6.7 to −1.5)] and the probiotic [−5.2 (95% CI, −7.7 to −2.7)] group. Likewise, the total score was different between probiotic and placebo groups, favoring a lower score when probiotic was used [placebo group: −7.1 (95% CI, −12.5 to −1.7); probiotic group: −11.6 (95% CI, −16.8 to −6.5)].

### 3.5. Safety

Nine participants (6 in the placebo group and 3 in the probiotic group) reported 20 adverse events (17 mild, 2 moderate, and 1 severe). Seventeen of these adverse events (8 participants total: 5 placebo group and 3 probiotic group) were deemed as “possibly or probably related” to the study product, all of which were mild gastrointestinal-related events [constipation (*n* = 4), gas/flatulence (*n* = 4), bloating (*n* = 4), abdominal pain/cramping (*n* = 2), and diarrhea (*n* = 3)]. The severe adverse event was intermittent chest tightness, deemed unrelated to the study product. Overall, no adverse events were significantly associated with either treatment, such that the probiotic supplement was considered safe and well-tolerated

## 4. Discussion

Currently, there is a significant emphasis on research exploring natural alternatives to enhance brain function and mental health [2]. Since 2013, a novel subclass of probiotics called ‘psychobiotics’ has emerged. These psychobiotics were first defined as probiotics that, when ingested in appropriate quantities, produced positive psychiatric effects in psychopathology [30] and then updated to any exogenous influence (i.e., probiotics, prebiotics, dietary fiber) whose positive effects on mental health and well-being are microbially mediated [31]. Indeed, probiotics can influence the gut-brain connection, offering a natural solution to manage stress and outcomes of mental health and well-being, potentially also having a significant impact on certain mental health disorders [32]. Therefore, an increasing number of clinical studies have been conducted to prove the impact of probiotics on brain and mental health [2].

*Lb. rhamnosus* HN001 has been widely studied over the past 25 years. It has been shown that the *Lb. rhamnosus* HN001 strain has multiple benefits for gut health [12,33], immunity [11,34], eczema [13,35], Gut microbiota modulation [36], gestational diabetes [14,37], postpartum depression [15], and also mental health and social functioning [16]. Interestingly, the *Lb. rhamnosus* species is known to enhance the immune system, support digestive health, and may also improve mental and brain health, although the effect remains strain- and dose-dependent [38]. For instance, a study carried out by Severance et al. [39] showed that supplementation with *Lb. rhamnosus* GG and two Bifidobacterium strains over 12 weeks improved Positive and Negative Affect Schedule (PANAS) scores in patients with schizophrenia. Interestingly, the effect of probiotics was associated with the decrease of Candida albicans in the gut, an associated yeast species in schizophrenia, without affecting other symbiotic yeasts [39]. Moreover, another study showed that *Lb. rhamnosus* significantly improved depression and quality of life (QoL) in individuals who underwent cutaneous aortic intervention [40]. In another study, an intervention with *Lb. rhamnosus* UBLR58 combined with glutamine (250 mg) showed significant changes in DASS-21, PSS, and state trait anxiety inventory (STAI) scores in a 4-week intervention trial [41]. Although there were statistically significant outcomes in this short-term intervention, the probiotic strains used were previously selected based on their ability to convert glutamine to GABA [41].

While the impact of *Lb. rhamnosus* HN001 on mental health has previously been assessed in three different controlled clinical trials; the mental health questionnaires used in these studies were not primary outcome measures. The first study was conducted by Tay et al. [16], who investigated the effect of *Lb. rhamnosus* HN001, at a dose of 6 × 10^9^ CFU day^−1^, on lowering blood sugar in prediabetes patients (*n* = 26) as well as on improving their mental health over 12 weeks as a secondary outcome. Although the number of participants was low, the beneficial effect of *Lb. rhamnosus* HN001 on social functioning and anxiety was observed (*p* = 0.05 and 0.007, respectively) [16].

Additionally, a study by Slykerman et al. [15] included 423 women at 14–16 gestational weeks, randomized to receive either *Lb. rhamnosus* HN001 (6 × 10^9^ cfu day^−1^) or a placebo for the remaining duration of pregnancy and for six months of birth while breastfeeding [15]. Mothers taking *Lb. rhamnosus* HN001 reported significantly lower anxiety and depression scores post-partum compared with those taking a placebo [15]. However, in the present study, the PSS and OHQ results showed an improvement without reaching statistically significant results in the female subgroup. This could be related to the study design, given that the Slykerman et al. [15] study had used a larger sample size and longer intervention duration. Moreover, our study excluded pregnant women and used a different set of questionnaires from Slykerman et al. [15], which used (Edinburgh Postnatal Depression Scale and State Trait Anxiety Inventory 6 item version). In addition to that, our study had more drop out in the female compared to the male subgroups (Table 3). This may explain the differences in the results of this study.

Additionally, in a recent consumer experience study conducted in 2022, *Lb. rhamnosus* HN001 was consumed by 47 consumers who reported an increase in happiness total scores (OHQ score) by 72% and 79% after 30 and 60 days, respectively [17]). Together, these results offer valuable insights into the potential benefits of incorporating HN001 into consumer routines, particularly to enhance happiness levels.

In this study, there was no statistically significant difference in the total happiness score between the groups. At the end of the intervention, participants taking *Lb. rhamnosus* HN001 demonstrated an increase in happiness from baseline to the end of the study compared with the placebo group. Moreover, the categorized total happiness score distribution showed that the percentage of “not happy” participants at baseline who became “happy” by the end of the study was 10% higher in the probiotic group compared with the placebo group. To our knowledge, there have been no previous studies on the use of probiotics to increase happiness, although many studies have shown probiotic effects on decreasing stress, anxiety, and depression. The percentage of participants taking probiotics that reported happiness at the end of the study was consistent with the same percentage found in a previously conducted consumer research study (65% versus 72%, respectively). Participant inclusion criteria used in our present study included a range of PSS scores (≥14) that represent moderate to high-stress levels. In the majority of similar studies, only moderately stressed participants were selected [42,43]. Thus, our results could have been influenced by the proportion of participants with high-stress scores at baseline. Either an extended intervention duration or homogenous PSS levels at baseline may be advantageous for discerning statistically significant differences between HN001 and placebo groups in future studies.

Surprisingly, post-hoc analysis revealed that in men, *Lb. rhamnosus* HN001 significantly improved the total happiness score compared with placebo throughout the study (*p* = 0.014). Furthermore, the total happiness score increased from baseline to the end of the study in the *Lb. rhamnosus* HN001 group but not in the placebo group. Interestingly, a significant change occurred from day 14 to the end of the study (T2–T3), highlighting the time required for HN001 to start exerting its effect. This finding aligns with previous studies that indicate that a probiotic typically needs more than 4 weeks to improve mental health [42,43,44]. However, no significant changes were observed in the female subgroup. The difference observed between the female and male subgroups may be attributed to the fact that the female participants had a mean age of around 46 (P75th, 57), encompassing the premenopausal and menopausal phases. Although pre-and menopausal age were not firmly associated with psychological outcomes, Freeman et al. [45] found that menopausal hot flashes were significantly associated with stress, depression, and anxiety in menopausal women. Furthermore, it has been shown that women are at greater risk for psychological issues due to the combination of biological and social determinants [46]. As mentioned above, an extended intervention duration could provide additional insights into whether HN001 requires a longer period to manifest effects within this specific subgroup. This prolonged investigation would offer a more comprehensive understanding of the dynamics underlying probiotic efficacy among women at different life stages. Interestingly, in the female subgroup, the percentage of female participants in the *Lb. rhamnosus* HN001 group that reported a change from “not happy” to “happy” was 13% higher than that in the placebo subgroup (70% versus 57%, respectively). Thus, *Lb. rhamnosus* HN001 showed improvement in happiness in the female subgroup as well.

Similarly, *Lb. rhamnosus* HN001 also showed a decrease in perceived stress levels throughout the study compared with the placebo group, although this difference was not statistically significant. The distribution of the categorized perceived stress scores showed that the percentage of participants taking *Lb. rhamnosus* HN001, who reported lower stress levels at the end of the study, was higher than those in the placebo group (47% versus 39%, respectively). On the other hand, consistent with the happiness outcomes, the female subgroup showed non-significant changes in all analysis types. Even so, 40% of females in the HN001 group reported low stress at T2 compared with 33% in the placebo group. As found in the results in happiness outcome, the male subgroup showed a positive benefit for HN001, with a statistically significant interaction between the intervention group and study days (*p* = 0.043). Additionally, at the end of the study, 53% of males in the probiotic group reported a low stress level, compared with 45% in the placebo group.

The effect size observed in the post-hoc sex subgroup analysis was 0.23 for changes in happiness outcomes and 0.20 for changes in perceived stress levels between the HN001 and control groups. It should be noted that the value of effect sizes in psychological studies remains controversial. Some meta-analysis studies on anti-depressants, e.g., agomelatine, showed a variation in effect sizes between studies ranging from 0.18 to 0.26 [47]. Although such anti-depressants showed good clinical outcomes, the effect size was small on the Cohen scale [48]. Authors have reverted this small effect size to the positive outcomes found in the placebo group, up to 0.9 in some studies [49], which led to a lowering of the treatment effect size. On the other hand, the effect size found in agomelatine seems to be higher than other drugs in different health conditions, such as ACE inhibitors in preventing cardiovascular events and acute stroke (effect size = 0.16 and 0.11, respectively) [50].

Overall, the effect size of *Lb. rhamnosus* HN001 strain intake in increasing happiness and lowering the stress level in the male is considered a small effect size in the Cohen scale; However, it is still at the same effect level found for pure anti-depressant molecules such as pure ACE inhibitor drugs. As shown in many previous studies, the placebo group in this current study also showed an improvement in happiness, stress level, and other stress, anxiety, and depression outcomes, but at a lower level than the HN001 group. This may explain why there was no statistically significant difference between groups and sheds light on the psychological effect of placebo intervention [51,52,53], as well as the need for larger study sizes.

The effects of *Lb. rhamnosus* HN001 on mental health requires further investigation to clarify the underlying mechanisms. However, it has been shown that *Lb. rhamnosus* HN001 has a powerful immunity effect in different age ranges (infants, adults, and the elderly) [11,13,34,35]. Therefore, it is conceivable that the effect observed in this study may be linked to an immunomodulatory effect that resolved subclinical inflammation, which in turn reduced the hyperactivity of the hypothalamic-pituitary-adrenal (HPA) axis [54], as observed in patients with a PSS score of 14 or higher. A study by Huang et al. [18] showed that *Lb. rhamnosus* HN001, as a single strain or in combination with *B. animalis* subsp. lactis HN019 attenuated the depressive-like and anxiety-like behaviors induced by chronic unpredictable mild stress (CUMS) in rats. The authors investigated the underlying mechanism and found that *Lb. rhamnosus* HN001 improved the former conditions by modulating rats’ gut microbiota already affected by the induced CUMS, normalizing the blood levels of both neurotransmitters and inflammatory markers [18]. Furthermore, another study [19] showed that *Lb. rhamnosus* HN001 altered the brain gene expression in stress-sensitive Wistar Kyoto rats. In this study, *Lb. rhamnosus* HN001 altered the expression of the metabotropic glutamate receptor (Grm4) in the amygdala [19]. These preclinical studies, alongside human clinical results, provide additional evidence of the potential of *Lb. rhamnosus* HN001 strains to support mental well-being and its ability to improve the gut-brain connection in the host.

Overall, the results underscored the effect of HN001 on happiness, stress, anxiety, and depression levels, showing an improvement in the probiotic group, although the changes were not statistically significant. Lew et al. [43] showed significant changes in anxiety, but not depression scores, between a probiotic group (Lactobacillus plantarum P8, 2 × 10^10^ CFU day^−1^) and a placebo group using DASS-42 [43]. Similarly, the authors showed that there were no significant changes in PSS scores, which is consistent with our results. Additionally, the authors used the DASS-42 rather than the DASS-21, which is a more sensitive tool for detecting any slight changes in SAD segments. When comparing our DASS-21 and PSS outcomes, it should be noted that Lew et al. [43] recruited participants with only moderate stress levels as measured by the PSS-10. In another clinical trial using Lactobacillus plantarum 299v [44], the results showed that there were no significant changes in psychological questionnaires, including the PSS-10, after an 8-week intervention. However, blood biomarkers related to immunity and kynurenine metabolism showed a significant difference between the study groups. It is worth noting that the probiotic and placebo groups were taking SSRI drugs during the intervention [44]. On the other hand, Chong et al. [42] evaluated Lb. plantarum DR7 in a 12-week clinical trial, *n* = 111 preselected by moderate stress (PSS-10) at the baseline; the DASS-42 results showed a significant decrease in different DASS-42 segments [42]. Based on the adverse events reported in this study, *Lb. rhamnosus* HN001 strain was safe and tolerable, as previously shown by numerous clinical studies [55,56,57,58].

This study may have been limited by the short duration of intervention. A longer intervention time may be needed to achieve a significant difference between the two groups.

## 5. Conclusions

This study sheds light on the impact of probiotics on happiness. *Lb. rhamnosus* HN001 showed promising results in this current controlled clinical trial, consistent with a previous open-label consumer study. We observed an improvement in total happiness and PSS scores over time after the 4-week intervention. Interestingly, the sex subgroup analysis showed statistically significant differences in both total happiness and PSS scores over time, emphasizing the need for larger and longer intervention studies to reach statistical significance, as well as the need for subgroup analyses to investigate sex-specific effects of probiotic intervention outcomes.

## Figures and Tables

**Table 1:** Participant characteristics.

Parameter	Statistic/Category	Overall *n* = 120	Group	
			Placebo *n* = 59	Probiotic *n* = 61
Age (years)	Mean (SD)	46.0 (15.1)	46.6 (15.8)	45.3 (14.5)
	Median (min, max)	48.0 (18.0, 78.0)	48.0 (21.0, 78.0)	48.0 (18.0, 72.0)
	P25, P75	33.0, 57.0	33.0, 57.0	33.0, 57.0
Sex	Female	60 (50.0%)	30 (50.8%)	30 (49.2%)
	Male	60 (50.0%)	29 (49.2%)	31 (50.8%)
Race	Black/African American	16 (13.3%)	6 (10.2%)	10 (16.4%)
	Multiracial	4 (3.3%)	3 (5.1%)	1 (1.6%)
	Other	4 (3.3%)	3 (5.1%)	1 (1.6%)
	Prefer Not To Specify	3 (2.5%)	3 (5.1%)	0 (0)
	White	93 (77.5%)	44 (74.6%)	49 (80.3%)
Time zone	Central	111 (92.5%)	55 (93.2%)	56 (91.8%)
	Eastern	4 (3.3%)	2 (3.4%)	2 (3.3%)
	Mountain	3 (2.5%)	2 (3.4%)	1 (1.6%)
	Pacific	2 (1.7%)	0 (0)	2 (3.3%)

SD, standard deviation; min, minimum; max, maximum; P25, 25th percentile; P75, 75th percentile.

**Table 2:** Psychological outcomes.

Variable	Placebo Mean (SD) N = 56	Probiotics Mean (SD) N = 57	Mean Difference (95% CI) at 27 Days	*p*-Value	Time Treatment Interaction *p*-Value
Happiness					
Baseline; T0	106.6 (22.0)	107.1 (18.7)			
14 days; T1	112.7 (22.6)	116.2 (22.1)	4.9 (−13.0 to 3.2)	0.23	0.61
27 days; T2	116.5 (22.5)	121.8 (22.1)			
Perceived Stress Scale					
Baseline; T0	24.6 (5.2)	24.0 (5.6)			
14 days; T1	17.7 (7.5)	17.8 (6.3)	1.6 (−0.9 to 4.2)	0.20	0.13
27 days; T2	16.6 (7.1)	14.9 (6.8)			
DASS (Stress)					
Baseline; T0	18.9 (9.4)	19.6 (8.8)			
14 days; T1	14.5 (9.1)	15.0 (9.7)	−0.3 (−3.5 to 3.0)	0.87	0.97
27 days; T2	12.8 (9.0)	13.1 (8.9)			
DASS (Anxiety)					
Baseline; T0	7.7 (7.9)	9.3 (9.1)			
14 days; T1	5.3 (7.6)	6.7 (8.3)	−0.6 (−3.3 to 2.2)	0.69	0.61
27 days; T2	5.0 (6.7)	5.6 (8.5)			
DASS (Depression)					
Baseline; T0	11.5 (10.1)	12.8 (10.1)			
14 days; T1	9.5 (9.5)	9.3 (9.3)	0.8 (−2.2 to 3.8)	0.59	0.4
27 days; T2	8.5 (8.2)	7.6 (8.4)			

SD, standard deviation; CI, confidence interval.

**Table 3:** Post-hoc sex group analysis.

Variable	Placebo Mean (SD)	Probiotics Mean (SD)	Mean Difference (95% CI) at 27 Days	*p*-Value	Time Treatment Interaction *p*-Value
Female participants					
	*n* = 28	*n* = 26			
Happiness					
Baseline; T0	103.6 (18.6)	104.2 (18.5)			
14 days; T1	109.0 (19.4)	117.1 (20.9)	−4.0 (−14.3 to 6.3)	0.44	0.19
27 days; T2	118.3 (17.6)	122.3 (22.1)			
Perceived Stress Scale					
Baseline; T0	26.4 (5.2)	25.5 (5.4)			
14 days; T1	19.3 (6.4)	18.3 (6.6)	1.7 (−1.7 to 5.1)	0.32	0.87
27 days; T2	17.0 (5.6)	15.3 (7.5)			
Male participants					
	*n* = 29	*n* = 30			
Happiness					
Baseline; T0	109.5 (24.9)	109.6 (18.8)			
14 days; T1	116.5 (25.2)	115.5 (23.5)	−6.0 (−18.7 to 6.8)	0.35	0.014 *
27 days; T2	114.7 (26.8)	121.3 (22.4)			
Perceived Stress Scale					
Baseline; T0	22.7 (4.4)	22.5 (5.4)			
14 days; T1	16.0 (8.2)	17.3 (6.1)	1.7 (−2.1 to 5.5)	0.38	0.043 *
27 days; T2	16.1 (8.5)	14.5 (6.1)			

SD, standard deviation; CI, confidence interval. An asterisk indicates a significant difference.

## Data Availability

The raw data supporting the conclusions of this article will be made available by the authors upon request.

## Supplementary Materials

The following supporting information can be downloaded at https://www.mdpi.com/article/10.3390/nu16172936/s1, Figure S1: Model-derived total happiness score estimate; Figure S2: Categorized total happiness score; Figure S3: Model-derived estimates of total happiness score within biological sex; Figure S4: Categorized happiness score by sex subgroup; Figure S5: Model-derived estimates of total PSS score (a) and of total PSS score within biological sex (b); Figure S6: Categorized PSS scores; Figure S7: Categorized PSS distribution by sex subgroup.
