# Supplementary material for: Effects of Lacticaseibacillus rhamnosus HN001 on Happiness and Mental Well-Being: Findings from a Randomized Controlled Trial
Source: Nutrients. 2024 Sep 2;16(17):2936. doi: 10.3390/nu16172936 (PMC11397133; doi:10.3390/nu16172936)
Supplement: Supplementary file 1 [file nutrients-16-02936-s001.zip › nutrients-3182046-supplementary.pdf]

Supplementary Materials

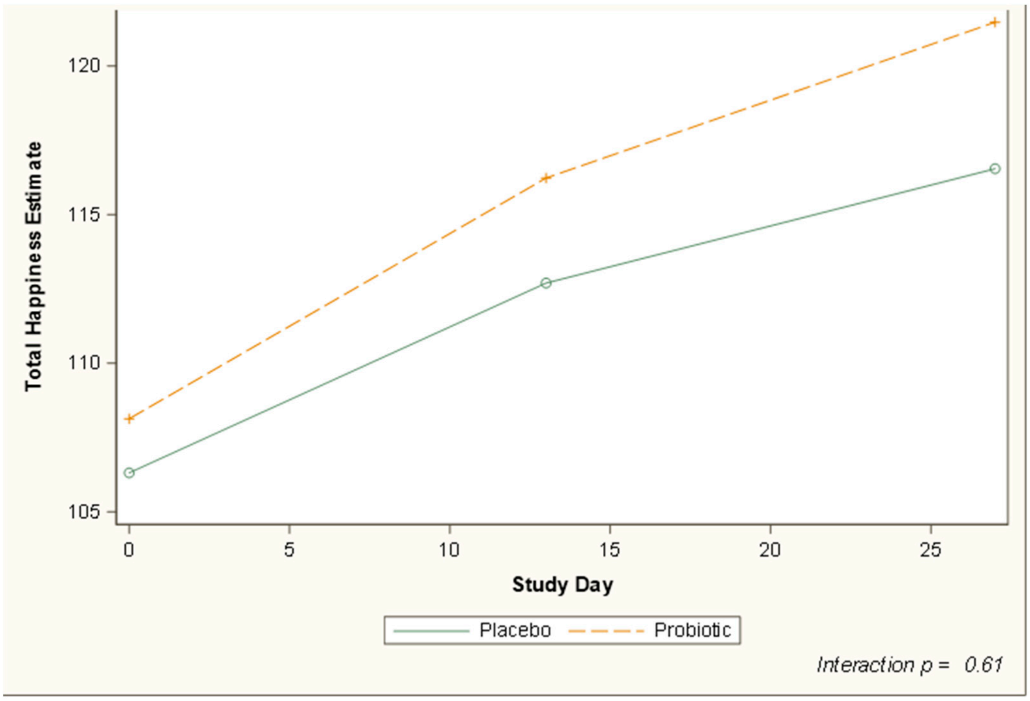

Figure S1 Model-derived total happiness score estimate

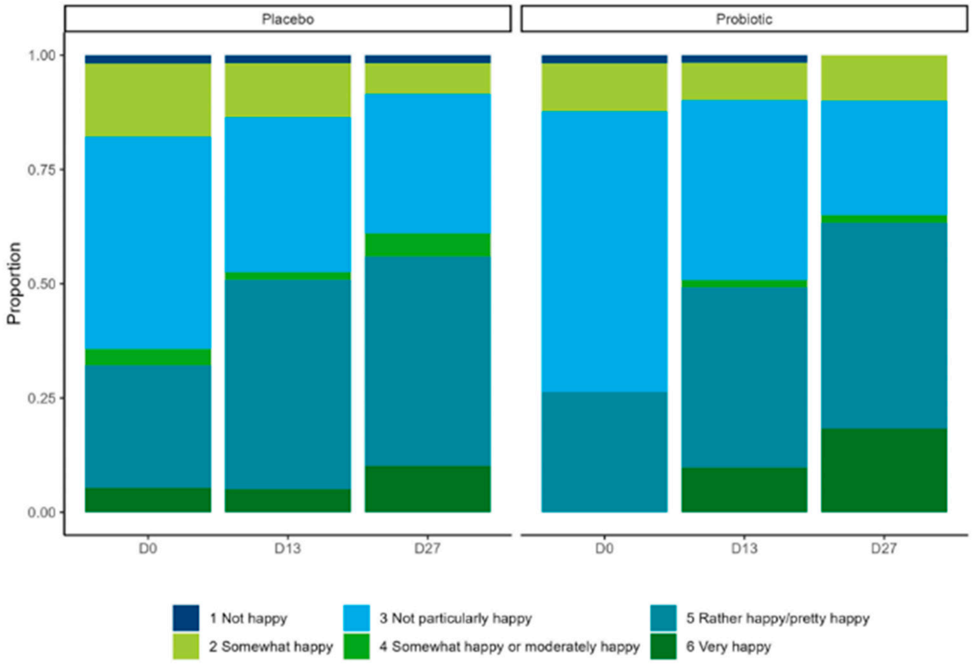

Figure S2 Categorized total happiness score

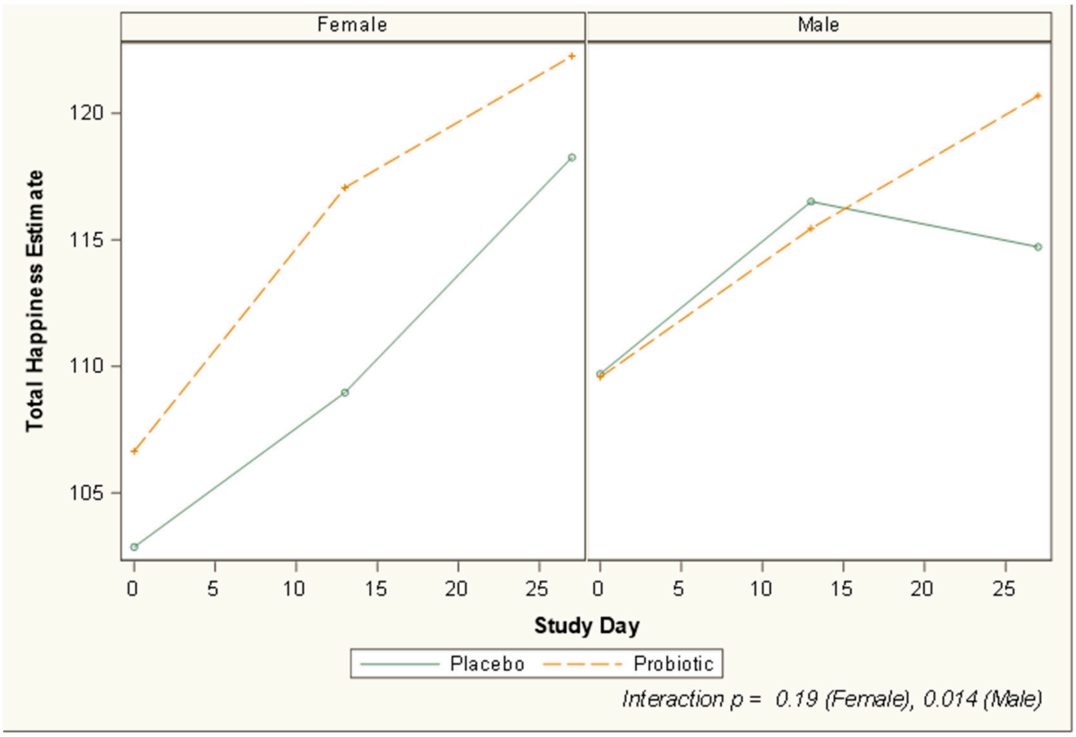

Figure S3 Model-derived estimates of total happiness score within biological sex

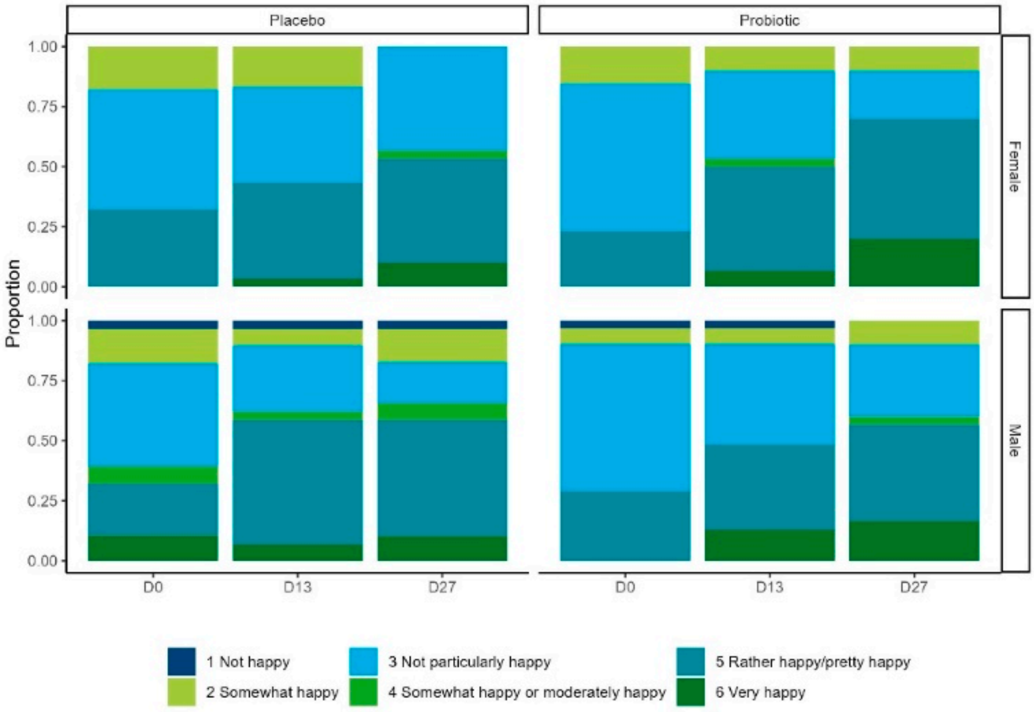

Figure S4 Categorized happiness score by sex subgroup

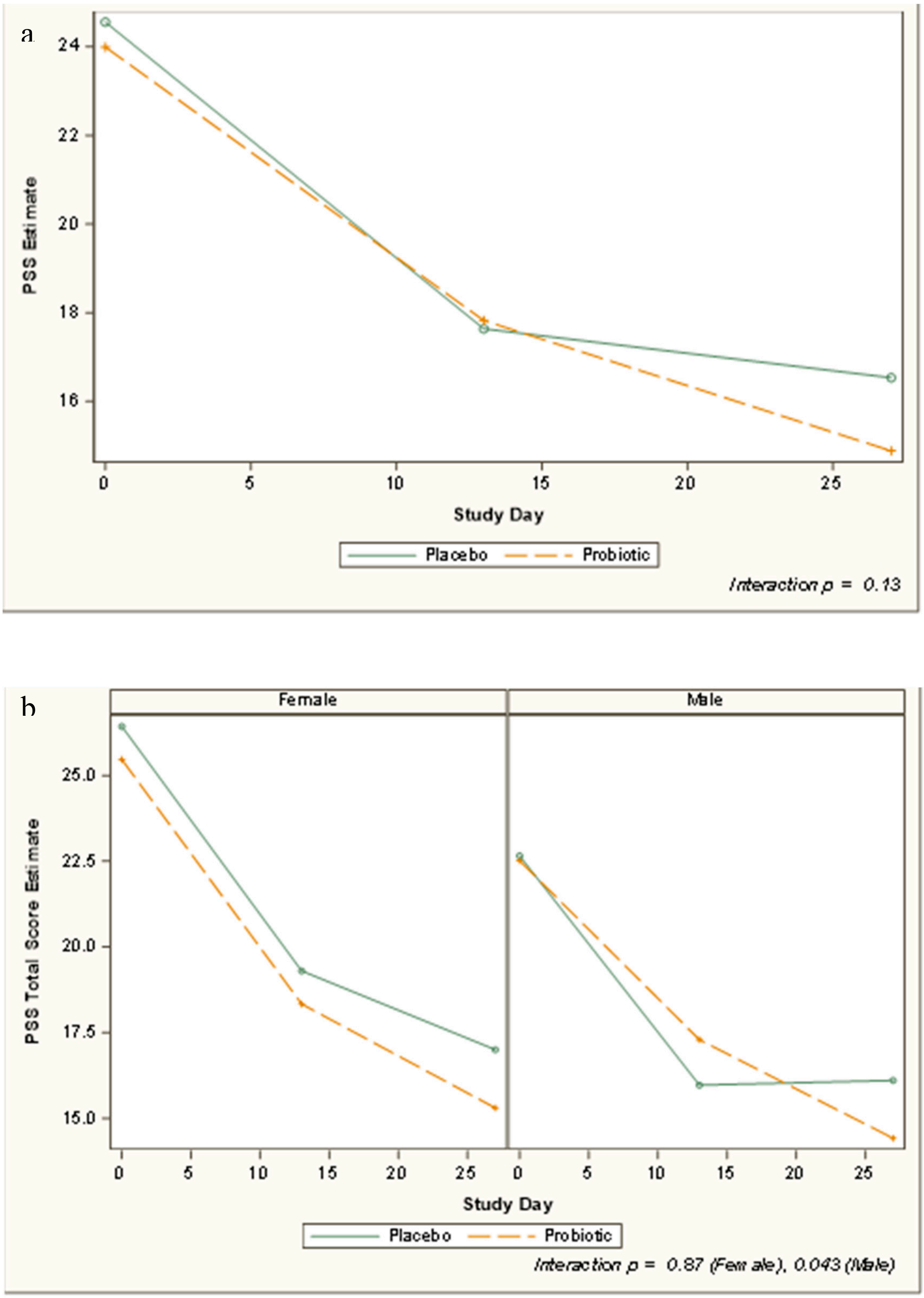

Figure S5 Model-derived estimates of total PSS score (a) and of total PSS score within biological sex (b)

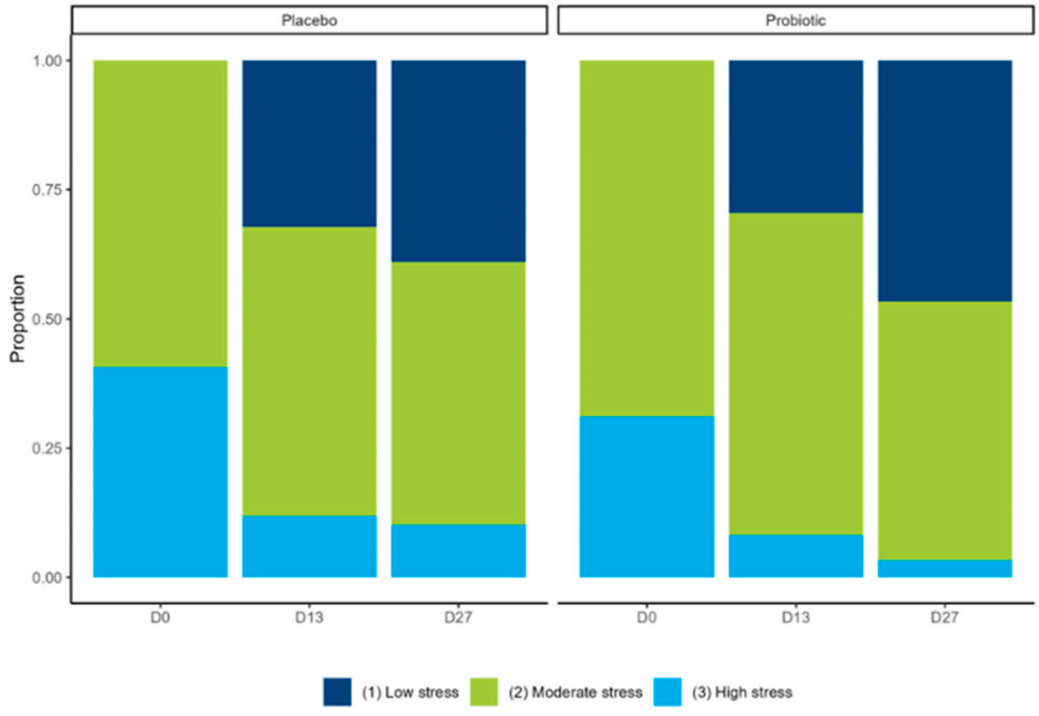

Figure S6 Categorized PSS scores

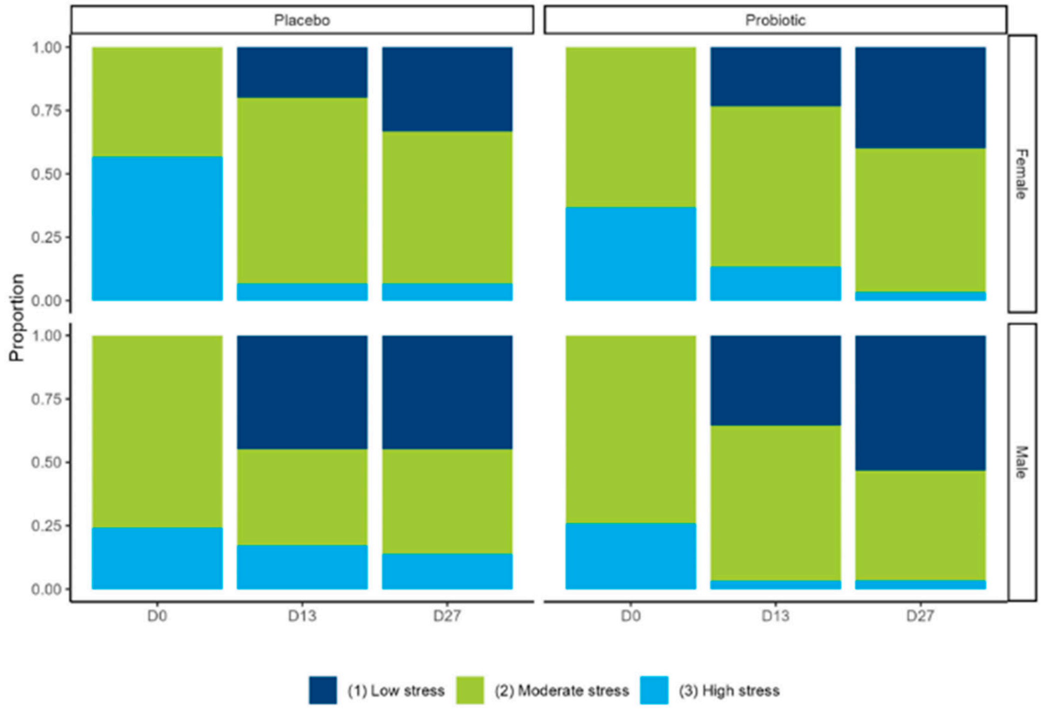

Figure S7 Categorized PSS distribution by sex subgroup
